# Arboreal camera trapping sheds light on seed dispersal of the world’s only epiphytic gymnosperm: *Zamia pseudoparasitica*

**DOI:** 10.1002/ece3.8769

**Published:** 2022-03-24

**Authors:** Claudio M. Monteza‐Moreno, Lilisbeth Rodriguez‐Castro, Pedro L. Castillo‐Caballero, Edgar Toribio, Kristin Saltonstall

**Affiliations:** ^1^ 56292 Department for the Ecology of Animal Societies Max Planck Institute of Animal Behavior Konstanz Germany; ^2^ 56292 Smithsonian Tropical Research Institute Panama City Panama; ^3^ Estación Científica COIBA‐AIP Ciudad del Saber Panamá City Panama; ^4^ Santiago Panama

**Keywords:** camera trap, Epiphytism, Northern olingo, Panama, seed dispersal, Zamia

## Abstract

Epiphytic lifestyles have evolved independently in ecologically, morphologically, and taxonomically diverse plant species. Although this adaptation is widespread among angiosperms, it is only known to have arisen in a single gymnosperm species, *Zamia pseudoparasitica* (Cycadophyta). *Zamia pseudoparasitica* is endemic to the mountains of Western Panama, and little is known about the ecology of this unusual cycad. Here, we provide the first report of a potential seed disperser of *Z*. *pseudoparasitica*. Between late October 2019 and March 2020, we conducted arboreal camera trapping at three sites along the Talamanca Cordillera in Western Panama, yielding an accumulated survey effort of 271 camera days. Weekly direct observations were also performed using handheld binoculars at one site. Arboreal camera trapping revealed at least seven mammal species that visit this epiphytic cycad. At all three sites, the Northern olingo (*Bassaricyon gabbii*) was seen visiting individuals of *Z*. *pseudoparasitica* repeatedly, both while cones were closed and after they had opened. We estimated the time‐varying intensity of the visits throughout our sampling and used mixed models to compare the length of visits when cones were closed versus when they were open. Both duration and time‐varying intensity of visits increased after cones had opened and we documented Northern olingo removing and carrying away seeds. We also observed predation by the yellow‐eared toucanet (*Selenidera spectabilis*) which picked and destroyed mature *Z*. *pseudoparasitica* seeds. These results suggest that the Northern olingo could be an important seed dispersal agent for this rare epiphytic gymnosperm.

## Graphical abstract

We conducted arboreal camera trapping in three sites along the Talamanca Cordillera to assess for potential seed dispersal agents of *Zamia pseudoparasitica*, which is the only known gymnosperm species to be epiphytic and it is endemic to the mountains of Western Panama. We found out that the potential arboreal mammal potentially acting as the seed dispersal agent is the Northern olingo.

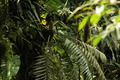

## 1 INTRODUCTION

Seed dispersal is a key biological process for maintaining populations of plant species (Levin et al., 2003; Traveset et al., 2014). Seeds can be dispersed by wind, gravity, water, and animals (Traveset et al., 2014). Regardless of the mechanism, seed dispersal depends on a variety of conditions such as the shape and size of the fruit (Howe & Miriti, 2004; Jansen et al., 2004), and in the case of animal seed dispersal, it may also depend on adaptations and animal behavior (Herrera, 1985; Stiles, 2000). For some plant species, there can be multiple animal species that contribute to their seed dispersal (Asquith et al., 1997; Bonaccorso et al., 1980; Lomáscolo et al., 2010). In other cases, seeds are dispersed by a single animal species. For example, the seeds of *Solanum dasyphyllum*, *S*. *aculeastrum* (Mwanza, 1995), *Omphalocarpum mortebani*, and *Mammea africana* (Yumoto et al., 1995) are known to be dispersed only by forest elephants, while *Trewia nudiflora* depends entirely on horned Asian rhinoceros to disperse its seeds (Dinerstein & Wemmer, 1988). However, while animal‐mediated seed dispersal is well‐known among angiosperms, we know relatively little about dispersal of gymnosperms seeds by animals, especially for cycads.

Globally, there are 358 recognized cycad species (Calonje et al., 2013), of which nearly 65% are endangered or near extinction due to habitat fragmentation and illegal trafficking for horticulture (Fragnière et al., 2015). The family Zamiaceae comprises 236 species (Calonje et al., 2019). Members of the genus *Zamia L*. are strictly Neotropical and are widely considered the most ecologically and morphologically diverse cycads (Morrone, 2014; Norstog & Nicholls, 1997). To disperse their seeds, this ancient genus, which originated around 68.3 Ma (Calonje et al., 2019), has presumably depended upon several species of animals that over time have evolved and become extinct. Despite cycads being the most common tropical gymnosperms, many aspects of their ecology remain unstudied (Clark & Clark, 1988), including which animal species serve as seed dispersal agents.

Among gymnosperms, epiphytism is only known to have arisen once, in *Zamia pseudoparasitica* (Yates, 1854), an endemic plant found in the mountains of Western Panama (Stevenson, 1993; Taylor et al., 2012). Epiphytism, *sensu stricto*, is a lifestyle adopted by nonparasitic plants, and it has appeared a number of times within angiosperms (Benzing, 1990; Zotz & Bader, 2009). In epiphytic angiosperms, seeds are commonly dispersed by wind, although some epiphytes produce fruits that can be dispersed by birds, bats, and primates (Granados‐Sánchez et al., 2003). Here, we explore potential seed dispersers of *Z*. *pseudoparasitica*. We used camera traps to conduct arboreal surveys of the species of animals visiting individuals of *Z*. *pseudoparasitica* in three montane rainforests of Western Panama. Previous studies have suggested that bats (Bell‐Doyon & Villarreal, 2020; Stevenson, 1993; Taylor et al., 2012) and toucans (Taylor et al., 2012) could act as seed dispersers for *Z*. *pseudoparasitica*, as seen in other cycad species (Hall & Walter, 2013; Tang, 1989); however, these studies lack empirical support.

## 2 METHODS

### 2.1 Study sites

**FIGURE 1:**
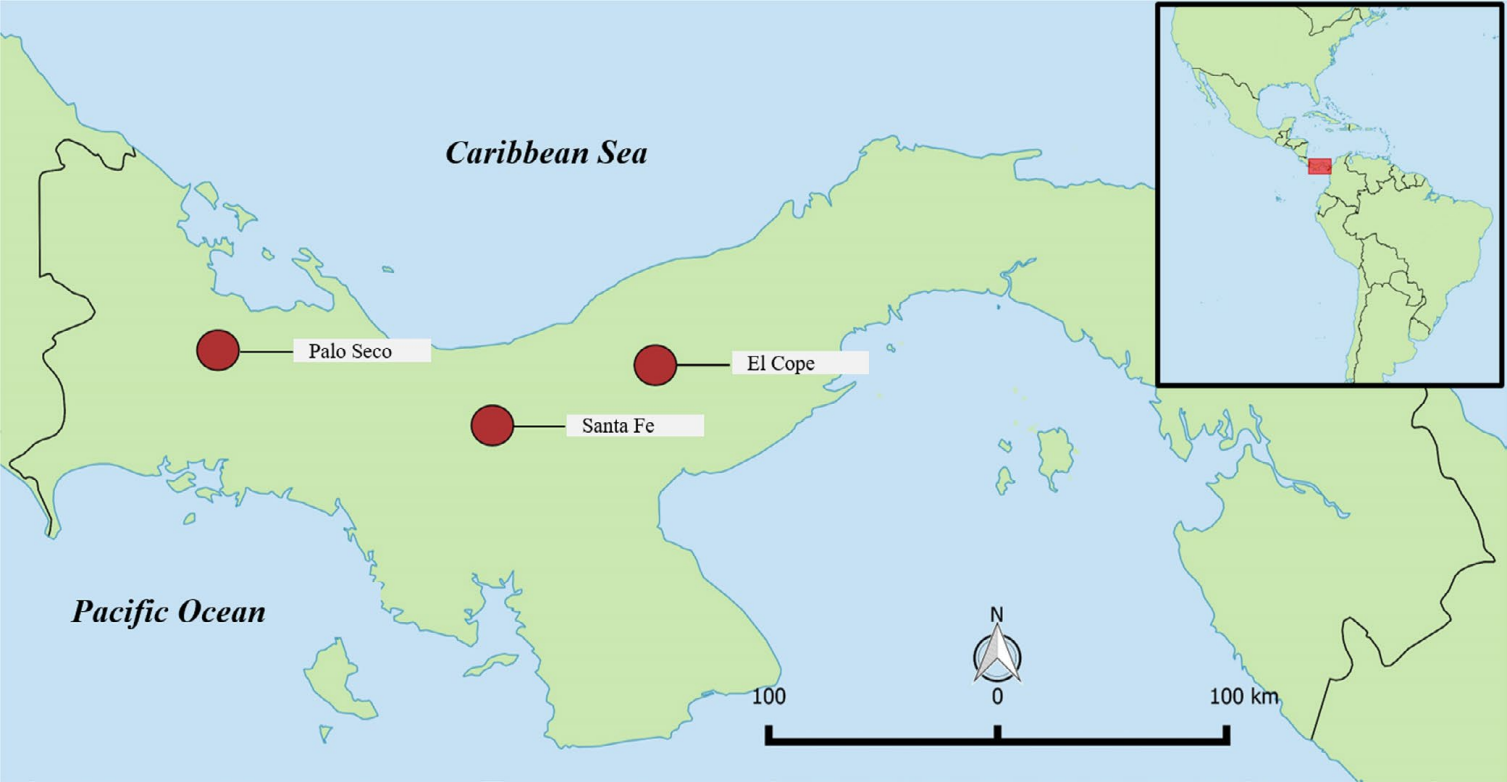
Map of the Republic of Panama, showing the location of the three study sites: Palo Seco, Santa Fe, and El Cope. Map was produced in QGIS 2.18.14

We conducted this study in the Cordillera of Talamanca in Western Panama (Figure 1). The cordillera is characterized by cloud forests and has a distinct flora and fauna (González‐Maya et al., 2009), with a high number of endemic species, including birds (Stattersfield, 1998), amphibians (Lips et al., 2003), and plants (Davis et al., 1997). We collected data in three protected areas in Western Panama: Palo Seco National Park (Palo Seco), Santa Fe National Park (Santa Fe), and Omar Torrijos Herrera National Park (hereafter, El Cope).

### 2.2 Study species

**FIGURE 2:**
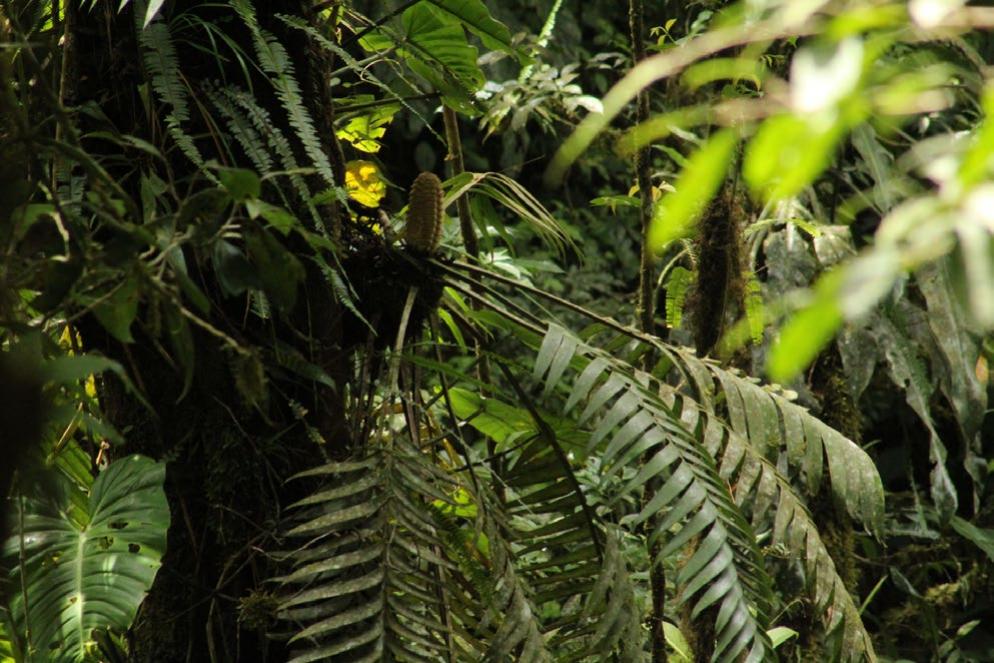
Individual of *Zamia pseudoparasitica* from Santa Fe National Park, Panama

*Zamia pseudoparasitica* (Figure 2; Yates, 1854) is endemic to the montane rainforests of Panama, with an altitudinal distribution range from 50 to 1,000 m.a.s.l. on the Atlantic side of the Cordillera (Stevenson, 1993). It is the only known epiphytic gymnosperm in the world (Taylor et al., 2012) and can be found at heights of 7–20 m, attached to large tree trunks, and nestled in lower forks of large canopy trees. Like all cycads, *Z*. *pseudoparasitica* is dioecious and produces either a single cream or tan‐colored polleniferous strobilus (male cone) that grows up to 50 cm long and 4 cm wide or a single yellow‐green or tan‐colored ovulated strobilus (female cone) that can reach 50 cm in length and 12 cm in diameter. Compared with its congeners, *Z*. *pseudoparasitica* produces very distinctive seeds that are covered in a thick yellow mucilaginous sarcotesta that emits a peculiar sour odor upon ripening. Seeds can grow up to 2.5 cm in length and 1.5 cm in diameter and are attached in pairs to the megasporophyll. *Zamia pseudoparasitica* strobili and seeds are among the largest in the genus (Stevenson, 1993).

### 2.3 Data collection

To carry out this pilot assessment, we conducted arboreal camera trapping. At each of the three study sites, we located one female *Z*. *pseudoparasitica* plant with a closed cone growing on a host tree that could be accessed using a single‐rope climbing technique (i.e., a healthy tree with branches capable of holding the weight of the climber). In Santa Fe and El Cope, we found host trees near trails inside the forest, whereas the host tree in Palo Seco was located on a small farm, approximately 35–50 m away from the forest edge. Single camera traps (Reconyx HP2X Hyperfire 2, Reconyx, Inc., WI, USA) were mounted at each site, between 15 and 20 m above the ground and facing the cones of each *Z*. *pseudoparasitica* at a distance of 1.5–2 m. Camera traps remained in the field from late October 2019 until March 2020, yielding an accumulated survey effort of 271 camera days. We programmed the camera traps to take 10 photographs upon each trigger without delay between triggers (observations). We also set the cameras to take one (time‐lapse) photograph at midnight and midday to differentiate wildlife absence from a malfunction of the cameras.

We annotated the wildlife species on the images using Reconyx MapView Professional (Version: 3.7.2.2) and followed Reid (2009) for species identification. For every animal we observed in proximity to the cones, we recorded the species name, time and duration of the visit, and animal behavior: for example, whether or not it inspected the cones, attempted to collect seeds, collected seeds and/or scent‐marked.

Additional direct observations of 15 *Z*. *pseudoparasitica* individuals were made from the ground along different trails at Santa Fe. These opportunistic observations were made during the day (usually in the morning) using handheld binoculars (10 min/individual/visit), at least once a week over the same period as the camera trapping survey, meaning that revisits happened every 2–3 weeks. Due to logistical constraints, we could only conduct these direct observations in Santa Fe.

As we recorded a high number of visits by the Northern olingo, further analyses were conducted to differentiate the interactions of this species with *Z*. *pseudoparasitica* cones when cones were closed versus when they were open. We estimated the time‐varying intensity of visits using the R package Functional Data Analysis (version 5.1.4; Ramsey et al., 2020). Further, we used the time stamps from subsequent camera images to calculate the exact length in seconds of each Northern olingo visit. We used the R package glmmADMB (Skaug et al., 2014) to compare the lengths of the visits when the cone was closed versus when it was open. We fitted a Gaussian mixed model to the visit durations (natural log transformed), including site as a random effect in the model, and generated mean estimates of duration when the cone was closed versus opened (Monteza‐Moreno et al., 2022).

## 3 RESULTS

**FIGURE 3:**
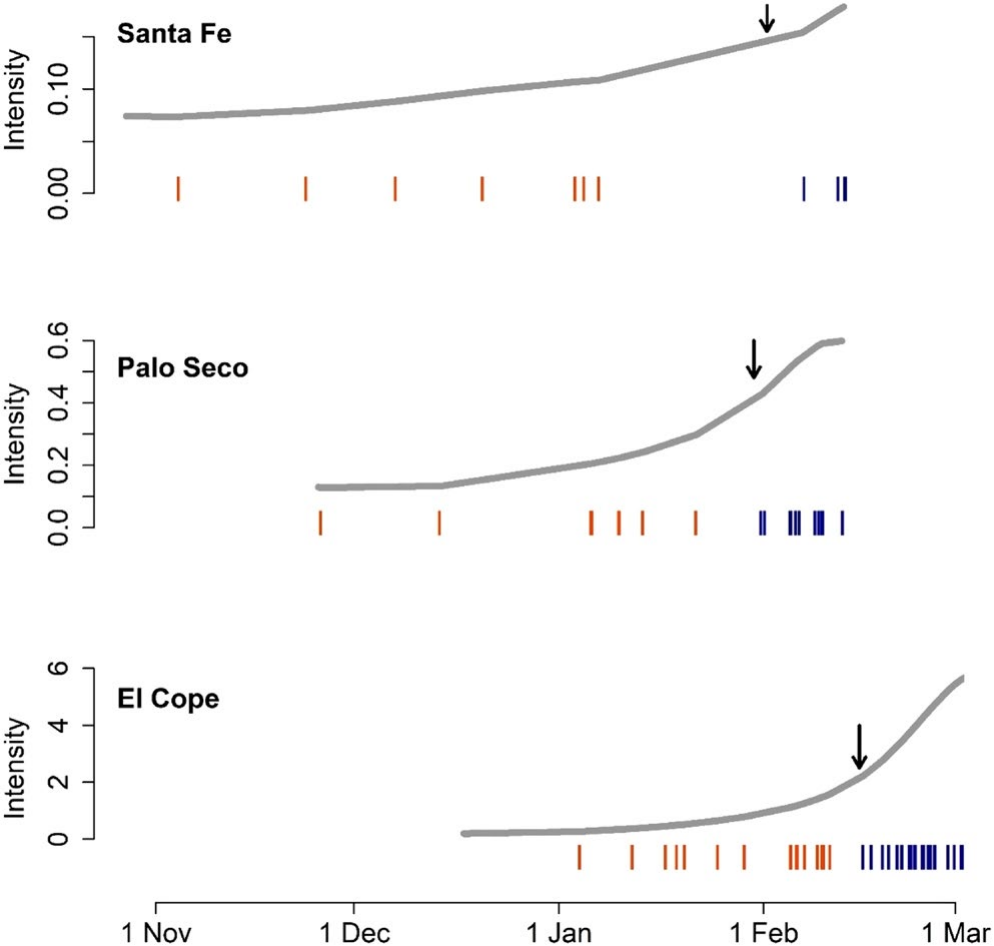
Time‐varying intensity of visits by northern olingo to cones of *Zamia pseudoparasitica* over our sampling period of Nov. 2019–March 2020. Observed visits are colored coded, where orange indicates visits when the cone was closed and blue when open. The arrows indicate the date the cones opened

All cones on our three study plants were full size but immature in November 2019, and the time‐lapse feature in our camera trap images documented a progressive maturation and opening of the cones. From the camera trap observations, it appears that animals cannot remove seeds when the cones are closed. In two of the focal *Z*. *pseudoparasitica*, cone opening occurred by the end of January and in the third individual, around mid‐February (Figure 3), which is mid‐way through the dry season in Panama. Although animals visited the cones while closed, the camera traps showed no animal intervention in the opening process of the cones.

**FIGURE 4:**
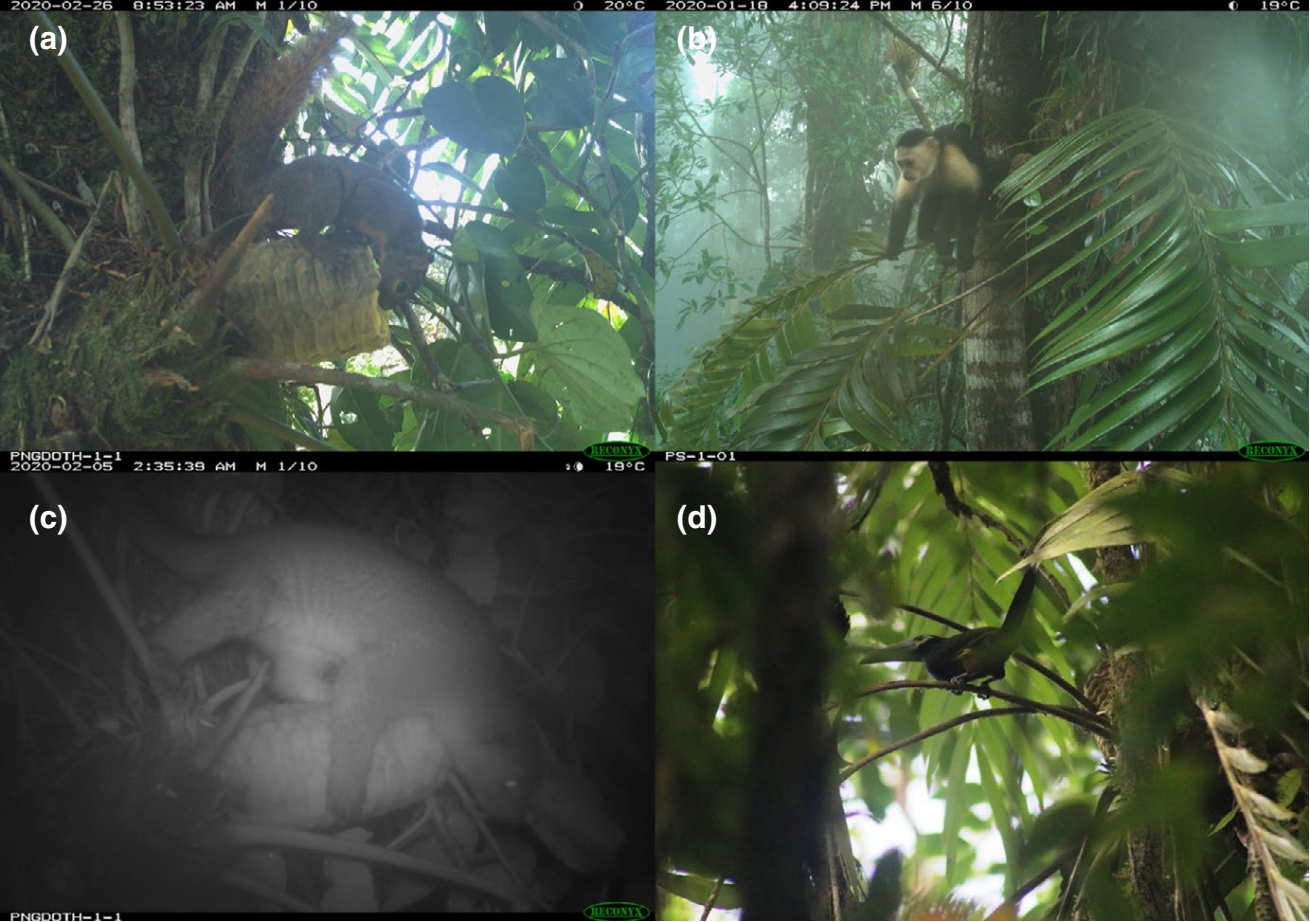
Some of the species observed in individuals of *Zamia pseudoparasitica*: (a) dwarf squirrel, (b) white‐faced capuchin monkey, (c) kinkajou, and (d) yellow‐eared toucanet

Our camera traps detected seven species of mammals on branches occupied by *Z*. *pseudoparasitica* (Table 1; Figure 4), but species differed in how they interacted with *Z*. *pseudoparasitica*. Northern tamandua, dwarf squirrel, and Robinson's mouse opossum were only detected once, passing next to the plants without stopping or interacting with the cones. White‐faced capuchin monkeys, Central American woolly opossum, and kinkajous inspected immature cones, but did not collect seeds. Both woolly opossums and kinkajous were also seen licking around the base of immature cones. Northern olingo was the only mammal species observed to visit *Z*. *pseudoparasitica* at all three study sites, and our camera traps captured multiple instances of them inspecting and collecting seeds from *Z*. *pseudoparasitica* cones (Video S1). Northern olingo also appeared to scent‐mark the cones and/or the branches adjacent to the plants. After cones had opened, Northern olingos’ visits, but not those of other species, became more clustered in time and nearly twice as intense (Figure 3). Our model estimated that the mean duration of a visit by Northern olingo was 6.4 s (95% CI: 3.0, 13.6) when the cones were closed; and increased to 23 s (95% CI: 11.1, 47.6) when the cones were open. Once opened, Northern olingo removed between 1 and 4 megasporophylls (2–8 seeds, respectively) per visit.

**TABLE 1:** Number of observations of wildlife near *Zamia pseudoparasitica* at three sites in the Cordillera of Talamanca in Western Panama and summary of the seed dispersal behavior per animal species

Scientific name	Common name	Palo Seco	Santa Fe	El Cope	Seed dispersal behavior
*Bassaricyon gabbii*	Northern olingo	13	7	44	Collected seeds
*Caluromys derbianus*	Central American woolly opossum	–	17	2	No seed collected
*Potos flavus*	Kinkajou	–	4	2	No seed collected
*Tamandua mexicana*	Northern tamandua	–	1	–	No interactions
*Marmosa robinsoni*	Robinson's mouse opossum	–	1	–	No interactions
*Cebus capucinus imitator*	White‐faced capuchin monkey	1	–	–	No seed collected
*Microsciurus spp*.	Dwarf squirrel	–	–	1	No interactions
*Selenidera spectabilis*	Yellow‐eared toucanet	–	1	–	Seed picked/destroyed

In the 3 months of our study, weekly observations of 15 *Z*. *pseudoparasitica* plants from the ground at Santa Fe noted only one vertebrate interaction. A yellow‐eared toucanet (*Selenidera spectabilis*) was observed picking and destroying the seeds of a mature cone of *Z*. *pseudoparasitica* in late January 2020.

## 4 DISCUSSION AND CONCLUSIONS

Arboreal camera trapping proved to be a useful method for documenting wildlife that may play an important role in the dispersal of *Z*. *pseudoparasitica*. In 271 camera trap days of monitoring single cones of individual *Z*. *pseudoparasitica* plants at three different sites of Western Panama, we recorded four species of mammals interacting with the cones of *Z*. *pseudoparasitica* and three mammal species passing by without interactions. With the exception of the Northern tamandua, which primarily eats social insects (termites and ants; Patterson et al., 1992), all the species we recorded in the vicinity of *Z*. *pseudoparasitica* are considered to be pre‐dispersal seed predators (Bonaccorso et al., 1980; Janzen, 1971; Medellín, 1994).

Six of the seven mammals that we recorded in the proximity of our plants did not collect *Z*. *pseudoparasitica* seeds. White‐faced capuchin monkeys are known to eat a variety of seeds, including structurally protected seeds such as *Sterculia apetala* (Barrett et al., 2017) and also serve as seed dispersal agents for many plant species (Wehncke & Domínguez, 2007). However, in our single record of white‐faced capuchin monkey, the individual appears to only do a short inspection of the cone. Woolly opossum and kinkajous were frequent visitors and repeatedly inspected and even licked the base of the *Z*. *pseudoparasitica* cones but did not attempt to remove seeds. This is interesting because contact with the sap produced by the cones leads to moderate irritation of the skin in humans (LRC, pers. observations). Previous studies have found cycads contain chemical compounds that might result in neurotoxic effects (Brenner et al., 2003; Rivadeneyra‐Domínguez & Rodríguez‐Landa, 2014) but the chemical makeup of *Z*. *pseudoparasitica* tissues and its effects on animals is unknown at this time.

Arboreal camera trapping revealed that the Northern olingo (*Bassaricyon gabbii*) was the only species that regularly visited and carried away intact seeds from *Z*. *pseudoparasitica* cones. The Northern olingo is an arboreal, nocturnal, and frugivore, with an estimated home range of 23.5 ha (Kays, 2000), which occurs in mature forests up to 2,000 m.a.s.l. (Prange & Prange, 2009). Northern olingos have previously been reported to eat fleshy fruits from a variety of tree species in lowland forests (Kays, 2000), none of which have been reported in the montane forests that we surveyed (Condit et al., 2010). From our camera trap images, we observed that Northern olingo collected between one and four megasporophylls (two to eight seeds, respectively) and stored them in their mouths before moving away from the *Z*. *pseudoparasitica* plant. However, due to the nature of our data collection methodology, we were unable to determine the fate of these seeds. While it is likely that many of the *Z*. *pseudoparasitica* were consumed, Northern olingo may also drop seeds after eating the sarcotesta and/or cache some proportion for future consumption, suggesting that this neotropical procyonid might be an important seed disperser for *Z*. *pseudoparasitica*.

Northern olingo regularly visited both closed and opened *Z*. *pseudoparasitica* cones, and in all three study sites, the visits became more frequent and 3.5 times longer after cones had opened (Figure 3). It appears that Northern olingo may monitor the status of this food resource and our photographs showed attempts to remove seeds by biting cones while they were still closed. Once cones opened and the seeds became available, Northern olingo returned to exploit them and invested more time per visit. It is possible that the frequent recaptures of Northern olingo on our camera traps were an incidental by‐product of their placement. For example, if the plants we monitored happened to be located at a territorial boundary, the frequent visits we observed could have arisen from intraspecific competition. We did observe Northern olingo appearing to scent‐mark the cones and/or the branches adjacent to the *Z*. *pseudoparasitica*. However, with the limited data currently available, it is not possible to distinguish between intentional visits to the plants for feeding or routine territorial marking behaviors. We are also unable to determine whether the visits we documented are attributable to a single individual at each site, or if multiple Northern olingos visited and exploited each of the cones we monitored. However, we find it unlikely that we would have selected *Z*. *pseudoparasitica* located on Northern olingo territorial boundaries three times, purely by chance, and our observation of more frequent and longer visits to the *Z*. *pseudoparasitica* plants after the cones opened suggests that feeding behavior is more likely.

The yellow‐eared toucanet was the only bird species observed picking seeds and destroying them on site. Previous work has considered toucans to be potential seed dispersers for Zamia species (Taylor et al., 2012); however, while toucans may move seeds away, this does not explain the epiphytic lifestyle of *Z*. *pseudoparasitica* as they might be excreting seeds in their feces anywhere in the forest rather than storing them in the canopy.

Previous studies have suggested that frugivorous bats may disperse *Z*. *pseudoparasitica* seeds (Bell‐Doyon & Villarreal, 2020; Stevenson, 1993; Taylor et al., 2012), as seen in *Dyssochroma viridiflorum*, an epiphytic species with seeds that do not exceed 1 cm in size (Sazima et al., 2003). Although monitoring of bats using camera traps is uncommon, some studies have demonstrated the utility of camera traps for recording the presence of bats (i.e., at roosts and caves; Baker, 2015; Mohd‐Azlan et al., 2010). However, our camera trapping did not obtain any observations of bats at the cones of *Z*. *pseudoparasitica*, which is perhaps not surprising due to the difficult maneuvering needed to remove the structurally hard seeds from the cones. Thus, our data do not support previous speculations that bats are seed dispersal agents of *Z*. *pseudoparasitica*.

As cycads are known for having numerous toxic chemicals (Brenner et al., 2003; Rivadeneyra‐Domínguez & Rodríguez‐Landa, 2014), it is possible that a limited number of wildlife can consume or disperse *Z*. *pseudoparasitica* seeds. The mechanism and efficiency of seed dispersal by Northern olingo are still unclear. Northern olingo may either be an accidental dispersal agent, swallowing the whole seed to absorb sugar compounds found in the sarcotesta, or alternatively caching the seeds of *Z*. *pseudoparasitica* for future consumption. The latter is a behavior that has been reported to benefit a variety of plant taxa (Brodin, 2010; Smith & Reichman, 1984), including other gymnosperms (Vander Wall, 1995, 2003; Vander Wall & Balda, 1981). In fact, seeds of gymnosperms that are cached are reported to have considerable germination success (Tomback et al., 2005). As *Z*. *pseudoparasitica* has a strictly epiphytic lifestyle (Stevenson, 1993; Taylor et al., 2012), it is unlikely that passive dispersal from animals dropping seeds is their primary means of spreading to new locations because seeds would likely fall to the ground due to their size and gravity. However, if seeds are cached in the canopy, they would be more likely to end up in favorable locations for germination and establishment, that is, in medium and large‐sized bryophytes, tree branch joints, or surfaces available between various canopy structures.

Our identification of the Northern olingo as a seed disperser for *Z*. *pseudoparasitica* is necessarily tentative, given our limited sampling, and particularly, the relatively short deployments of our camera traps after the cones opened. Additional studies following cones over the course of a year and for multiple years will help to unravel the dispersal biology of this unusual cycad. While other species may consume and disperse *Z*. *pseudoparasitica* seeds, it is also possible that selective pressures are constraining other species of mammals, but favoring Northern olingo, from manipulating *Z*. *pseudoparasitica* seeds.

The interaction between Northern olingo and *Z*. *pseudoparasitica* provides a fascinating system to investigate co‐adaptations and evolutionary interactions between a plant and its seed dispersers, which may improve our understanding of the lifecycle of this rare and unique epiphytic gymnosperm.

## CONFLICT OF INTEREST

The authors declare no conflict of interest.

## AUTHOR CONTRIBUTIONS

**Claudio M. Monteza‐Moreno:** Conceptualization (supporting); Data curation (supporting); Formal analysis (lead); Funding acquisition (equal); Methodology (supporting); Resources (supporting); Software (lead); Validation (equal); Visualization (lead); Writing – original draft (lead); Writing – review & editing (lead). **Lilisbeth Rodriguez‐Castro:** Conceptualization (supporting); Data curation (supporting); Investigation (lead); Methodology (supporting); Project administration (supporting); Validation (equal); Visualization (supporting); Writing – original draft (supporting); Writing – review & editing (supporting). **Pedro L. Castillo‐Caballero:** Conceptualization (equal); Data curation (lead); Investigation (supporting); Methodology (supporting); Validation (equal); Visualization (supporting); Writing – original draft (supporting); Writing – review & editing (supporting). **Edgar Toribio:** Investigation (supporting); Resources (supporting); Visualization (supporting). **Kristin Saltonstall:** Conceptualization (equal); Funding acquisition (equal); Project administration (supporting); Resources (supporting); Supervision (lead); Validation (equal); Visualization (supporting); Writing – original draft (supporting); Writing – review & editing (supporting).

## Supporting information

Video S1

## Data Availability

Datasets and R scripts are available on: https://doi.org/10.17632/ttz7wtcv4t.2.
